# Generation of a fully erythromycin-sensitive strain of *Clostridioides difficile* using a novel CRISPR-Cas9 genome editing system

**DOI:** 10.1038/s41598-019-44458-y

**Published:** 2019-05-31

**Authors:** Patrick Ingle, Daphne Groothuis, Peter Rowe, He Huang, Alan Cockayne, Sarah A. Kuehne, Weihong Jiang, Yang Gu, Christopher M. Humphreys, Nigel P. Minton

**Affiliations:** 1 Clostridia Research Group, BBSRC/EPSRC Synthetic Biology Research Centre (SBRC), School of Life Sciences, The University of Nottingham, Nottingham, NG7 2RD UK; 2 Key Laboratory of Synthetic Biology, CAS Center for Excellence in Molecular Plant Sciences, Institute of Plant Physiology and Ecology, Shanghai Institutes for Biological Sciences, Chinese Academy of Sciences, Shanghai, 200032 China; 3 Present Address: School of Dentistry, College of Medical and Dental Sciences, University of Birmingham, Edgbaston, Birmingham B5 7SA UK; 4 Present Address: Deep Branch Biotechnology, NG8 1AA Nottingham, United Kingdom

**Keywords:** Pathogens, Microbial genetics

## Abstract

Understanding the molecular pathogenesis of *Clostridioides difficile* has relied on the use of *ermB*-based mutagens in erythromycin-sensitive strains. However, the repeated subcultures required to isolate sensitive variants can lead to the acquisition of ancillary mutations that affect phenotype, including virulence. CRISPR-Cas9 allows the direct selection of mutants, reducing the number of subcultures and thereby minimising the likelihood of acquiring additional mutations. Accordingly, CRISPR-Cas9 was used to sequentially remove from the *C*. *difficile* 630 reference strain (NCTC 13307) two *ermB* genes and *pyrE*. The genomes of the strains generated (630Δerm* and 630Δerm*Δ*pyrE*, respectively) contained no ancillary mutations compared to the NCTC 13307 parental strain, making these strains the preferred option where erythromycin-sensitive 630 strains are required. Intriguingly, the *cas9* gene of the plasmid used contained a proximal frameshift mutation. Despite this, the frequency of mutant isolation was high (96% and 89% for *ermB* and *pyrE*, respectively) indicating that a functional Cas9 is still being produced. Re-initiation of translation from an internal AUG start codon would produce a foreshortened protein lacking a RuvCI nucleolytic domain, effectively a ‘nickase’. The mutation allowed *cas9* to be cloned downstream of the strong P_*thl*_ promoter. It may find application elsewhere where the use of strong, constitutive promoters is preferred.

## Introduction

*Clostridioides difficile* (formerly *Clostridium difficile*^1^) is a Gram-positive, anaerobic, endospore-forming bacterium, which is the leading cause of antibiotic-associated diarrhoea worldwide. *C*. *difficile* associated infection (CDI) is characterised by a range of disease symptoms ranging from mild, self-limiting diarrhoea to the potentially fatal pseudomembranous colitis (PMC) and places a significant burden on healthcare facilities. The principle virulence factors are the two large, glucosylating toxins A and B^2^. Efforts to identify and characterise those other factors important in CDI have centred on genomic studies and the application of mutagenic gene tools^2^.

The first *C*. *difficile* genome sequence determined was that of the erythromycin (Em) resistant (^R^), outbreak strain, 630^3,4^. Its genome contained two sequence identical *erm*(*B*) genes, termed *erm1*(*B*) and *erm2*(*B*), which formed part of the mobilizable, non-conjugative transposon Tn*5398*^5^. To enable the use of Em^R^ genes as selectable markers in available gene tools, sensitive (^S^) variants of strain 630 were required. These were independently isolated by two laboratories using serial passage in antibiotic-free media. Mullany and co-workers (UCL, London, UK) performed 30 such subcultures to isolate 630Δ*erm*^6^, whilst the Rood laboratory (Monash, Australia) performed an undisclosed number of subcultures to generate 630E^7^. Both 630Δ*erm* and 630E possess an identical 2.4 kb deletion in Tn*5398* that entirely removed *erm2*(*B*) together with the gene encoding CD630_2008, a predicted plasmid-partitioning protein.

The availability of 630Δ*erm* and 630E allowed the use of ClosTron and allelic exchange tools which capitalised on the use of *ermB* genes to generate insertional^8^ or clean deletion mutants^9^. However, it became apparent that the phenotypic behaviour of 630 toxin mutants, for instance, was influenced by which Em^S^ strain was employed as the progenitor^2,10^. It subsequently transpired that during their derivation, multiple single nucleotide polymorphisms (SNPs) and Indels (Insertion/deletions) had accumulated within their genomes compared to the parental 630 strain^11^. Furthermore, phenotypic comparisons of 630Δ*erm* and 630E revealed the latter grew more slowly than the former and to a lower final cell density, produced lower toxin titres and was less virulent in the hamster model of *C*. *difficile* infection^11^.

It is likely that the ancillary mutants that arose in 630Δ*erm* and 630E were a consequence of the repeated subcultures needed to isolate a spontaneous Em^S^ strain. This could be avoided by direct deletion of *ermB* from the 630 genome. Whilst this can be relatively easily achieved through the use of allelic exchange in combination with counter selection markers^12^, the desired mutants may not be directly selected. Rather, a sequential process is used in which single crossover integrants are first selected on media supplemented with an appropriate antibiotic. Following their purification, these cells are then plated on selective media containing the necessary counter selective agent to identify the desired double crossover mutant^9^. One consequence of the necessary repeated passage through single cell, clonal selection steps is the increased probability of isolating SNPs and Indels. A more rapid route to mutant generation would be preferable.

The recent exemplification of CRISPR-Cas9 mutagenesis in clostridia^13–15^, including *C*. *difficile*^16,17^, offers the facility to directly select double crossover mutants in the absence of traditional counter selection markers. The associated reduction in the number of steps needed to isolate mutants should minimise the risk of isolating variants in which secondary mutations have arisen. To test this assumption we sought to implement our previously developed CRISPR-Cas9 system^14^ in *C*. *difficile* and recreate a strain 630 variant sensitive to Em. Our design took the opportunity to delete both copies of *ermB*, thereby reducing the risk of reversion to Em^R^. During the course of this work, we isolated a CRISPR-Cas9 variant (trCas9) which, in the configuration used, facilitated its deployment in mutant generation.

## Results

### Modularisation of a CRISPR-Cas9 vector

We have previously constructed a CRISPR-Cas9 vector for use in a number of industrially relevant clostridia, including *Clostridium ljungdahlii*^14^. As *C*. *difficile* is also an acetogen^18^, we based our vector on this system. To build in utility we first modularised the vector to conform to the standardised, pMTL80000 modular vector system in which the application module (between the *Sbf*I and *Asc*I restriction enzyme sites) became the components required for CRISPR-Cas9 genome editing. The CRISPR-specific components were divided into the following sub-modules; (A) nuclease, (B) guide RNA, and (C) editing template. Submodules A and B are flanked with unique 6–8 nucleotide restriction sites; *Sbf*I and *Xba*I for submodule A, and *Xba*I, and *Asi*SI for submodule B. The latter also contains a *Sal*I site between the guide RNA promoter and the seed region of the guide RNA, enabling high throughput assembly methods to rapidly exchange guide targeting loci. Due to submodule C containing relatively long (>1500 bp) sequences, the 8 nucleotide *Asi*SI and *Asc*I restriction sites were selected to reduce the likelihood of unwanted occurrence of recognition sequences within the homology arms that comprise the editing template.

**Figure 1:**
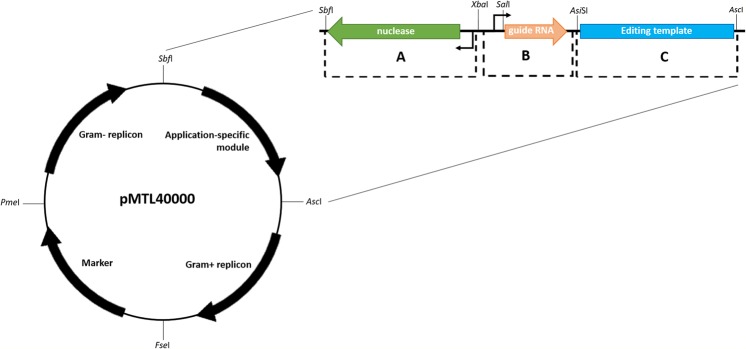
Modular vector design of pMTL40000, showing three CRISPR-specific sub-modules within the application specific module of the pMTL80000 vector series. Module A consists of a nuclease and promoter (reverse orientation) and is flanked by SbfI and XbaI. Module B consists of the RNA component(s) of the nuclease in module A, along with a promoter. This is flanked by XbaI and AsiSI, and contains an internal SalI site between the promoter and guide RNA. Module C consists of the homology cassette derived from the left- and right- homology arms of the gene of interest, and is flanked by AscI and AsiSI restriction sites.

Initially the vector pMTLcas-pta previously shown to generate a *pyrE* knockout in *C*. *ljungdahlii*^14^ was adapted to the modular format^19^. Early iterations of the vector were designed to incorporate an alternative restriction site, *Mau*BI, between submodules B and C, however, this was later substituted to *Asi*SI as commercially available *Mau*BI proved relatively inefficient in DNA cleavage (METHODS). The final pMTL40000 vector design can be seen in Fig. 1. For pMTL431511-CLAU-pyrE, the pCB102 Gram-positive replicon was used, along with the CatP selective marker, and ColE1 + *tra* Gram-negative replicon module. The CRISPR-specific application module comprises (A) P_*thl*_ controlling the expression of *cas9* RNA (B) the P_*araE*_ promoter controlling the expression of sgRNA containing the *pyrE* targeting seed region, and (C) the homology cassette consists of 1 kB regions immediately up- and down-stream of the *pyrE* gene.

### Implementation of the modular CRISPR vector in *C*. *difficile*

**Figure 2:**
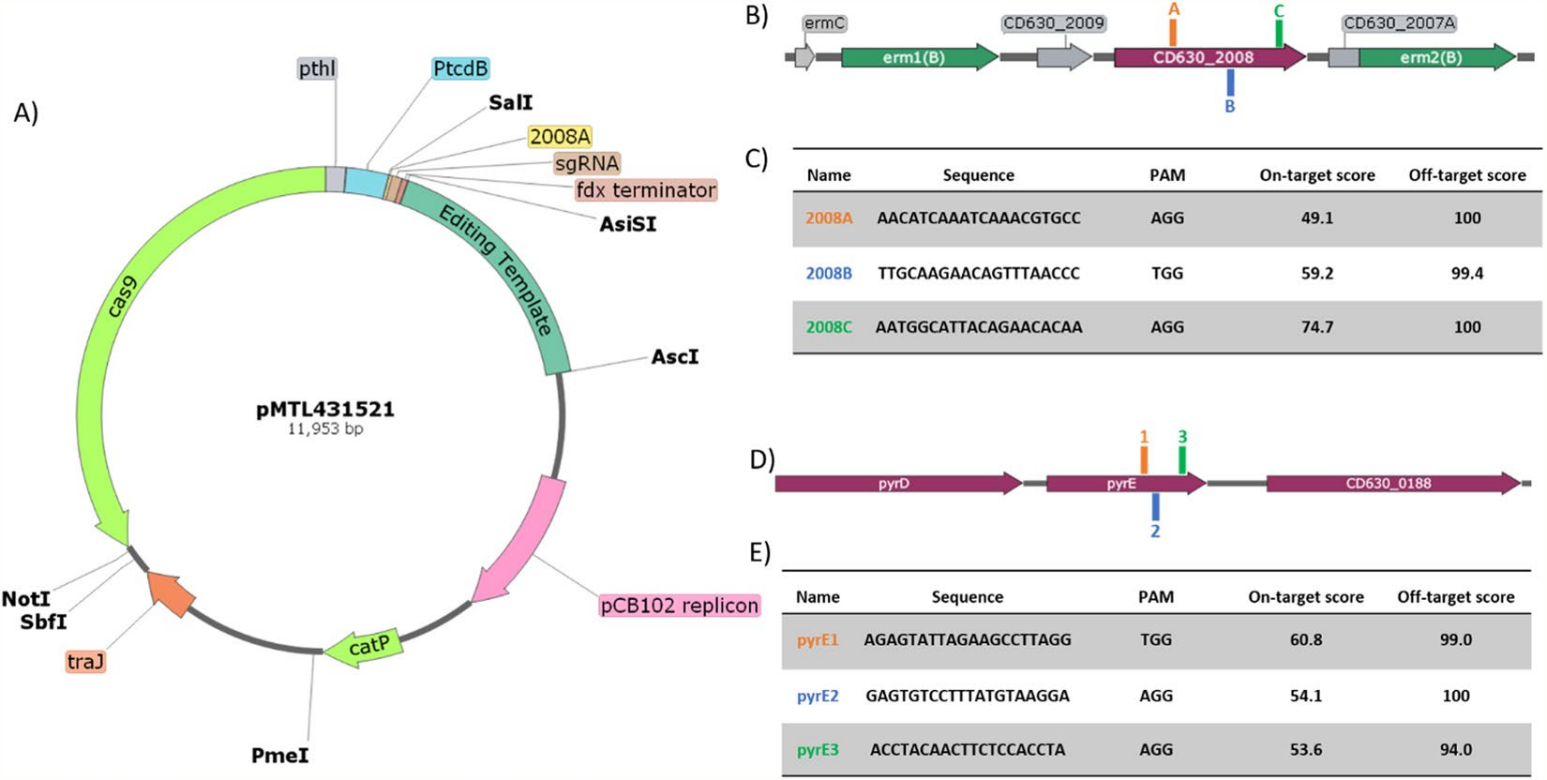
CRISPR-Cas9 genome editing vector and retargeting crRNA sequences. (**A**) Diagrammatic representation of the CRISPR-Cas9 vector developed in this study. The requisite CRISPR-Cas9 components, namely *S. pyogenes cas9*, under the control of the *C. acetobutylicum* thiolase promoter (P_*thl*_); an sgRNA component, consisting of a gRNA handle and crRNA (2008A) under the control of *C. difficile* toxin B promoter (P_*tcdB*_), and an editing template, containing upstream and downstream chromosomal regions flanking the deletion target site, cloned between *AsiSI* and *AscI* restriction sites. The plasmid backbone consists of the *E. coli*-clostridia shuttle vector pMTL83151. (**B**) Positions of 20-nt crRNA retargeting sequences within the region targeted for deletion from Tn*5398* labelled A–C, corresponding to the 2008A-C sequences listed in (**C**), along with on- and off-target scores provided by the Benchling CRISPR guide design tool. The protospacer-associated motif (PAM) for each crRNA sequence is also stated. Similarly, locations (**D**; not to scale) of the three crRNA retargeting sequences (**E**) present in the three CRISPR-Cas9 vectors utilised for truncation of 234 bp at the 3′-end of *pyrE*.

**Figure 3:**
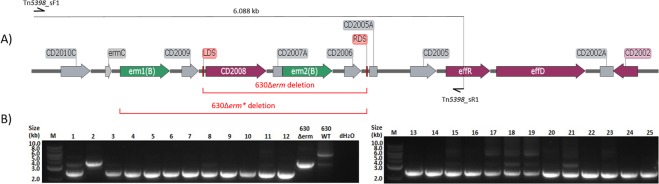
Colony PCR screening of putative 630Δerm* strains. (**A**) Diagrammatic representation of the 9.6 kb *C. difficile* 630 Tn*5398* mobilizable transposon sequence. The *erm* (**B**) genes are shown in green, other named ORFs shown in purple, and gene remnants and pseudogenes are shown in grey. Red brackets denote the genomic region deleted in 630Δerm, flanked by the left- (LDS) and right-deletion sites (RDS), and targeted for deletion in the generation of 630Δerm*. Schematic binding sites of screening PCR primers Tn5398_sF1 and Tn5398_sR1 (not to scale) and the size of the expected wild-type product are also shown. (**B**) Colony PCR screening of 25 putative 630Δerm* mutants using Tn5398_sF1 and Tn5398_sR1 primers. ‘M’ denotes DNA marker, ‘dH_2_O’ denotes negative control lane.

Following modularisation of the CRISPR vector the plasmid was adapted for use in *C*. *difficile*. The nuclease selected for submodule A remained the *cas9* gene from *Streptococcus pyogenes* under the control of the strong thiolase promoter from *Clostridium acetobutylicum* as it is known to function effectively in *C*. *difficile*^8,19^. For submodule B, the sgRNA cassette was placed under the control of the native *C*. *difficile* Toxin B promoter P_*tcdB*_, and an editing template for homologous recombination comprising approximately 1 kb up- and down-stream genomic regions flanking the desired 3.6 kb deletion region within Tn*5398* (Fig. 2A) made up submodule C. To thoroughly test our CRISPR-Cas9 genome editing plasmid, and since guide RNA prediction methods are in their infancy, we generated three CRISPR-Cas9 vectors targeting the CD630_2008 gene in Tn*5398*, with each containing a different 20 nucleotide crRNA sequence (Fig. 2B,C) from those identified using the Benchling CRISPR guide design tool^20^. These vectors, pMTL431521-CDF-2008A, pMTL431521-CDF-2008B and pMTL431521-CDF-2008C, were conjugated into our *C*. *difficile* 630 strain (CRG856) and the resulting thiamphenicol-resistant transconjugants which appeared after 48–72 hours were screened via colony PCR (Fig. 3). Of the 25 colonies screened, all but one generated a 2.44 kb-sized product signifying the deletion of both *erm*(*B*) genes from Tn*5398* with an editing efficiency of 96%. Eleven of these 25 PCR screens also showed bands at 6.088 kb and/or 3.682 kb indicating the presence of wild-type 630 or 630Δ*erm* Tn*5398* sequence, respectively, whilst thirteen appeared as pure mutants. From these thirteen pure mutants, three independently generated strains, one from each CRISPR-Cas9 vector, were carried forward for plasmid loss, with the subsequent thiamphenicol-sensitive strains designated as 630Δerm*.

### The *cas9* gene of pMTL431521 contains a frame shift

**Figure 4:**
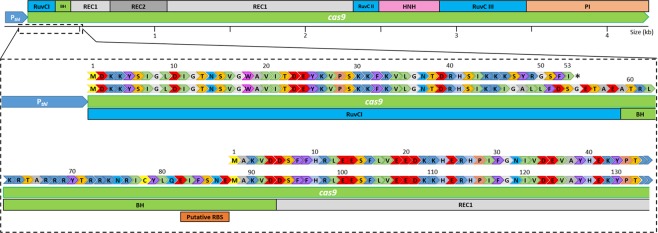
Diagrammatic representation of trCas9 sequence and annotated features. Top: Domain organisation within wild-type *S. pyogenes* Cas9. Bottom: Domain organisation and DNA sequence of our trCas9 and upstream thiolase promoter (P_*thl*_) from pMTL431511-CLAU-pyrE. The frameshift-generating adenine base insertion at position 130 within the cas9 RuvCI domain, which results in a truncated polypeptide of 53 amino acids, is indicated in capital letters. Putative ribosome binding site and downstream trCas9 ORF lacking 87 amino acids from the N-terminal end of Cas9 also indicated.

In parallel to the generation of the *erm*(*B*) deletion variants, the entire nucleotide sequence of the plasmids used were determined using appropriate primers and Sanger sequencing. Unexpectedly, a single adenine base insertion within a poly-A region starting at nucleotide position 130 was discovered which resulted in a frameshift mutation. This same single base insertion was also found (Fig. S1)  in the parental plasmid, pMTLcas-pta^14^. Closer inspection of the region of sequence affected identified an AUG start codon some 135 nucleotides 3′ to the frameshift that was preceded by a sequencing bearing some resemblance to a RBS which would allow a truncated Cas9 (trCas9) protein lacking 87 amino acids from the N-terminal end of Cas9 to be produced (Fig. 4). The deleted Cas9 domain encompasses a RuvCI nucleolytic domain. The trCas9 produced would, therefore, most likely represent a ‘nickase’ variant of Cas9 which produces single-strand nicks instead of DSBs. Cas9 nickase variants have been previously shown to be highly effective for genome editing in clostridia^13^.

### Generation of 630Δerm*Δ*pyrE*

**Figure 5:**
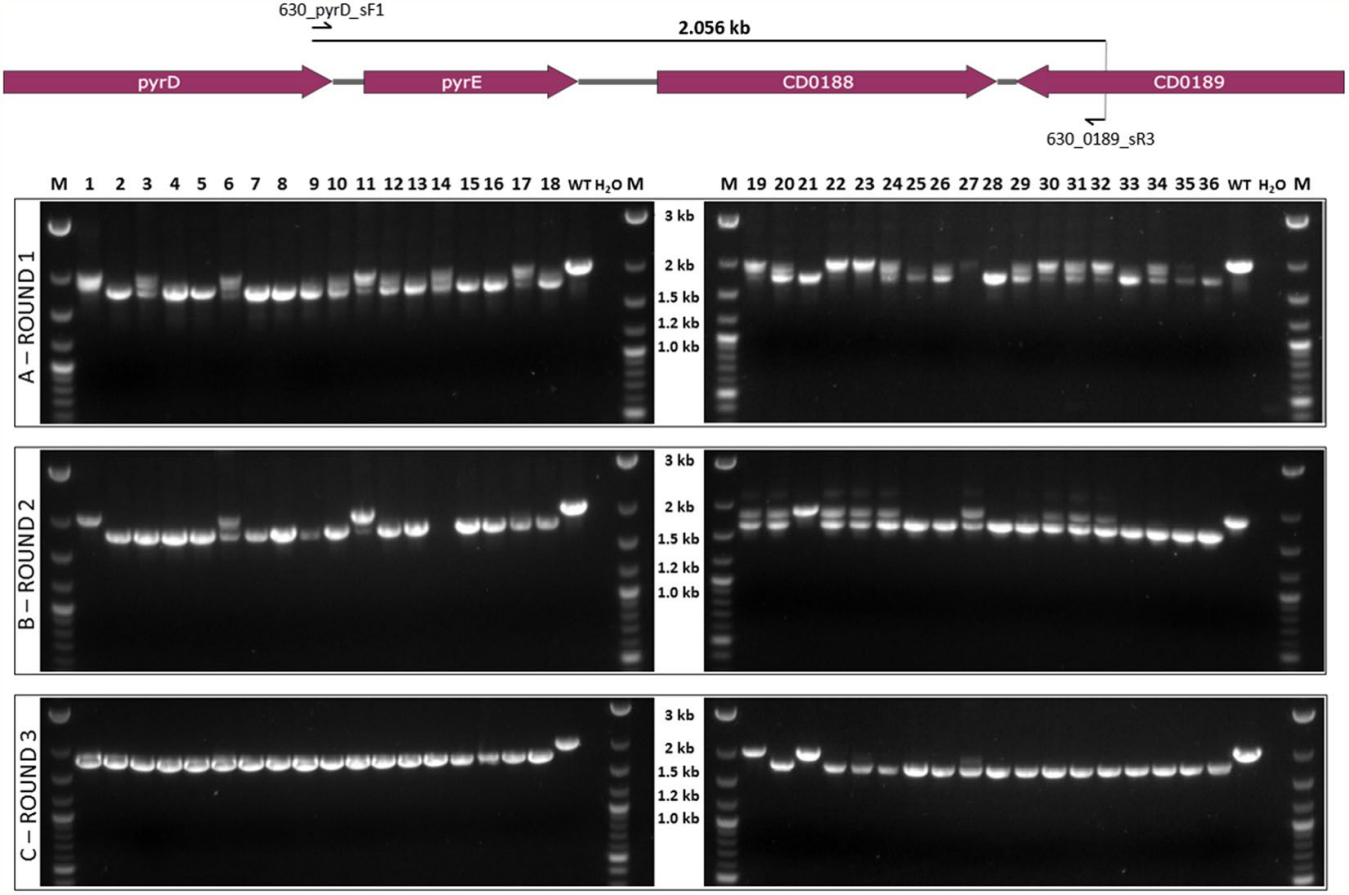
Colony PCR screening of putative 630Δerm*Δ*pyrE* strains. Top: Diagrammatic representation of the *C. difficile* 630 chromosomal region containing *pyrE*, including schematic binding sites of screening primers 630_pyrD_sF1 and 630_0189_sR3 (not to scale). Bottom: Agarose gel electrophoresis following colony PCR screens for the desired 234 bp truncation of *pyrE* following initial streaking of thiamphenicol resistant transconjugant colonies to purity (A) and two subsequent colony PCR screens following an additional one (B), or two (C), rounds of passaging on BHIS agar supplemented with thiamphenicol. ‘M’ denotes DNA marker, ‘dH_2_O’ denotes negative control lane.

Having successfully demonstrated that our CRISPR-Cas9 system was effective, despite the premature termination of the Cas9 polypeptide, we sought to further validate this system via the truncation of *pyrE* in each of our independent 630Δerm* strains. An editing template encompassing approximately 1 kb up- and down-stream of the 234 bp to be deleted from the 3′-end of the *pyrE* gene was constructed via SOE PCR and cloned into pMTL431521-CDF-2008A. HiFi assembly was again used to insert the three chosen crRNA sequences (Fig. 2D,E) from the 16 identified within the 234 bp target region, generating vectors pMTL431521-CDF-pyrE1, pMTL431521-CDF-pyrE2 and pMTL431521-CDF-pyrE3. These vectors were transferred into each of the independent 630Δerm* strains and 12 resulting thiamphenicol-resistant colonies from each conjugation were colony PCR screened for the desired deletion (Fig. 4). Whilst 32/36 colonies (89%) contained the 1.822 kb band indicative of this deletion, over half of these also contained a 2.056 kb band consistent with the wild-type *pyrE* sequence. Previous CRISPR-Cas9 studies have reported that the isolation of pure mutants following such mixed phenotypes from PCR screens can be improved via additional streaking of the mixed strains on selective media^14^. Therefore, we streaked all 36 colonies onto the same BHIS media supplemented with thiamphenicol twice more, performing colony PCR screens after each round of streaking (Fig. 5). After the first round of screening, 13/36 colonies were pure mutant with a further 19/36 displaying a mixed wild-type and mutant phenotype. The number of pure mutants obtained increased to 21 after one additional streak, and to 32 after a third round of screening, confirming the observations by Huang *et al*.^14^. Three independently generated pure *pyrE*-truncated mutants were sub-cultured to lose the plasmid and named CRG 630Δerm*Δ*pyrE* #1–3.

### Whole genome sequencing of CRG 630Δerm* strains

We next performed Illumina whole genome sequencing of our independent CRG 630Δerm* mutants, along with the parental CRG 630 strain, to assess whether any genomic variants had accumulated in these strains during CRISPR-Cas9 mutagenesis. Illumina reads from each strain were aligned to the 630 reference sequence AM180355.1 (Table S1). Whilst we were able to show that no additional mutations relative to the parental strain were present, we detected 17 variants present in each of our CRG 630 strains (parent and mutants) compared to the published reference genome. These variants consisted of 13 single nucleotide polymorphisms, three point insertions and a 21-nucleotide deletion from the CD630_26850 ORF resulting in an in-frame deletion of seven amino acids from the mature protein. Searches for conserved protein domains within the amino acid sequence of CD630_26850 identified C-terminal SpoIIE and serine phosphatase domains. This 21-nt internal deletion within CD630_26850 was confirmed via PCR and Sanger sequencing with flanking primers in our CRG 630 strain and its progeny, but was not observed in our 630Δ*erm* strain.

To investigate the effects of this 21-nt deletion, or indeed any of the other variants, on the ability of our CRG 630 strain and its progeny to form heat-resistant spores over five days we performed a rate of sporulation assay (Fig. S2). Development of heat-resistant CFU for CRG 630 and its progeny was lower, yet comparable, with that of 630Δ*erm* and this difference was not statistically significant. Furthermore, there was no significant difference between our CRG 630 strain and the 630Δerm* derivatives indicating the deletion of the 3.6 kb genomic region containing both *erm*(*B*) genes had no effect upon the proficiency of these strains to develop heat-resistant spores.

### Regeneration of 630Δerm* and 630Δerm*Δ*pyrE*

From these whole genome sequencing results it was clear that our aim of generating a new Em^S^ *C*. *difficile* 630 strain free from genomic variants had not been achieved due to an accumulation of ancillary mutants, and in particular the 21-bp deletion. Hence, we sought to repeat our CRISPR-Cas9 mutagenesis in a newly acquired reference strain of *C*. *difficile* 630 from the NCTC culture collection (NCTC 13307; ‘NCTC 630’). Accordingly, vectors pMTL431521-CDF-2008A, pMTL431521-CDF-2008B and pMTL431521-CDF-2008C were conjugated into NCTC 630 and, as before, three independent 630Δerm* strains lacking both *erm*(*B*) genes were obtained, one from each conjugation. Similarly, for the regeneration of 630Δerm*Δ*pyrE*, vectors pMTL431521-CDF-pyrE1, pMTL431521-CDF-pyrE2 and pMTL431521-CDF-pyrE3 were conjugated into the three 630Δerm* replicates and the desired truncation of *pyrE* observed in each case (Fig. S3).

#### Phenotypic characterisation of NCTC 630Δerm* and 630Δerm*ΔpyrE strains

**Figure 6:**
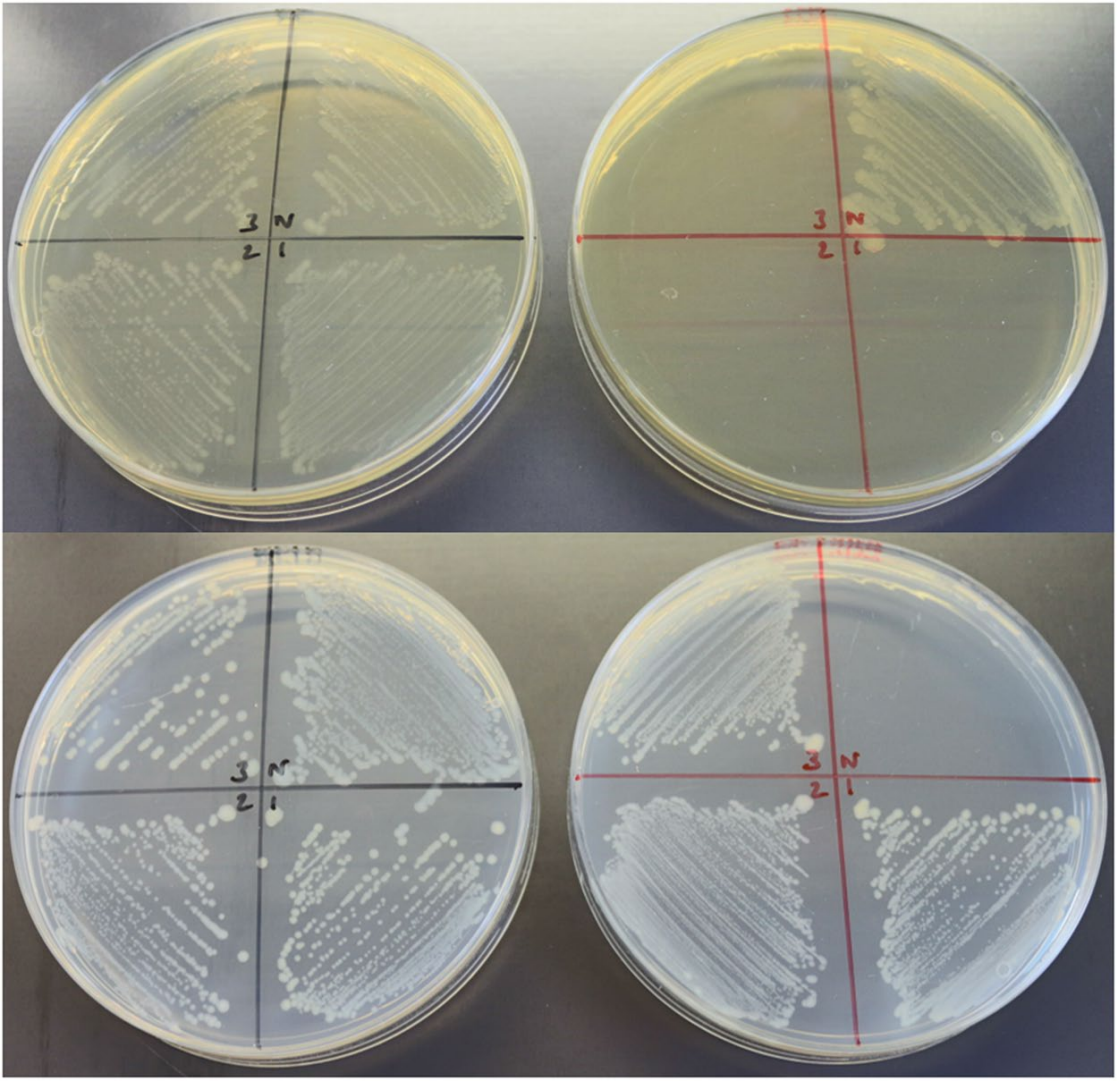
Phenotypic characterisation of NCTC 630 and 630Δerm*Δ*pyrE* strains. Growth of *C. difficile* strains NCTC 630 (N) and three independently generated 630Δerm*Δ*pyrE* mutants (#1–3) after 48 hours on BHIS (top left), BHIS supplemented with 10 μg.ml^-1^ Em (top right), *C. difficile* minimal media (CDMM) supplemented with uracil (bottom left) and CDMM supplemented with uracil and 5-flouroorotic acid (bottom right).

**Figure 7:**
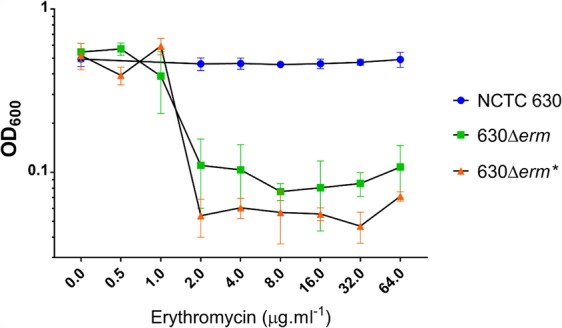
Determination of the minimum inhibitory concentration to Em of *C. difficile* 630 strains. Growth after 24 hours of *C. difficile* 630 strains in BHIS broth supplemented with various amounts of Em was determined via optical density measurements at 600 nm (OD_600_). Symbols represent mean values from three independent experiments and error bars indicate the standard deviation from the mean.

Having regenerated 630Δerm* and 630Δerm*Δ*pyrE* mutants in the NCTC 630 strain, we next confirmed the expected phenotypes of these strains by assessing growth after plating on appropriate media (Fig. 6). All three independent 630Δerm* mutants were unable to grow on BHIS media supplemented with 10 μg.ml^−1^ Em. Furthermore, only the three 630Δerm*Δ*pyrE* strains were able to grow on CDMM supplemented with uracil and 5-FOA, as in the parental NCTC 630 strain the product of the *pyrE* gene converts 5-FOA into the toxic compound 5-FUMP. To determine whether the deletion of both *ermB* genes from *C*. *difficile* 630 is sufficient to prevent the reversion to Em^R^ exhibited by 630Δ*erm*, we conducted serial passages of the parental 630 strain, 630Δerm* mutants and 630Δ*erm* in antibiotic-free, liquid media. After thirty such passages, neither 630Δ*erm* nor 630Δerm* exhibited a reversion to Em^R^ which would allow these strains to grow on BHIS media supplemented with 10 μg.ml^−1^ Em. We then determined the minimum inhibitory concentration of these strains to Em using the broth microdilution method (Fig. 7). The MIC of both 630Δ*erm* and 630Δerm* strains was determined to be 2 µg.ml^−1^, whilst the NCTC 630 strain was resistant up to 64 μg.ml^−1^, the highest Em concentration tested.

### Whole genome sequencing of NCTC 630Δerm* and 630Δerm*Δ*pyrE* strains

We then performed Illumina sequencing on our new NCTC 630 strain as well as each triplicate of the 630Δerm* and 630Δerm*Δ*pyrE* progeny. All genomic variants identified in these strains relative to the CP010905.2 reference sequence are outlined in Table S2 in Supplementary Information. The NCTC 630 strain was found to contain three genomic variants relative to the CP010905.2 reference sequence, consisting of a single adenosine base insertion in a non-coding region downstream of *argS*, and two non-synonymous SNPs in the *rpoB* and *perR* genes. Unsurprisingly, these variants were present in all of the subsequent mutants. Of the three triplicate 630Δerm*Δ*pyrE* strains, one (replicate #3) had accumulated no SNPs nor indels after the two rounds of CRISPR-Cas9 mutagenesis. Interestingly, in both rounds of mutagenesis this strain required the fewest passages to confirm the loss of the CRISPR plasmid. Meanwhile, replicates #1 and #2, which required up to four additional passages to confirm loss of the CRISPR, were found to have accumulated one and two SNPs, respectively. 630Δerm*Δ*pyrE* replicate #1 contained an additional non-synonymous SNP in *perR*, which encodes a peroxide-responsive repressor, whilst 630Δerm*Δ*pyrE* replicate #2 contained two additional non-synonymous SNPs; one in CDIF630_00864 encoding a putative lipoprotein and another introducing a premature stop codon in *eftA3*, which encodes an electron transfer flavoprotein alpha subunit. Each of these variants occurred during the first round of CRISPR-Cas9 mutagenesis to generate the 630Δerm* strains. Thus, no variants were accumulated in any of our triplicates during the second round of CRISPR-Cas9 genome editing to obtain the 630Δerm*Δ*pyrE* mutants.

We then aligned our NCTC 630 sequence data against other 630 and 630Δ*erm* reference sequences available from the NCBI database to see how this NCTC sequence compares to other 630 strains (Table S3). The NCTC 630 strain sequenced in this study is most similar to that sequenced by Riedel *et al*.^21^, (CP010905.2) who obtained their strain from the DSM culture collection (DSM 27543). As this DSM strain was acquired from the NCTC, the three variants we observe in our NCTC 630 strain most likely arose in the intervening time between transfer from the NCTC and sequencing of the DSM strain. Meanwhile, we observed 16 conflicts (13 SNPs, 3 point insertions) when comparing our NCTC 630 sequence with that of the 630 sequence from Sebaihia *et al*.^3^, only five of which are also present in both 630Δ*erm* sequences. This suggests that the first 630 strain sequenced (AM180355.1) had diverged from a progenitor strain from which 630Δ*erm* was generated, as we would expect all variants called in AM180355.1 to be present in the 630Δ*erm* sequences.

## Discussion

In this study, we used CRISPR-Cas9 genome editing to generate 630Δerm*, a fully Em^S^ variant of a *C*. *difficile* reference strain 630 (NCTC 13307), by removing both *ermB* genes present on the mobilizable transposon Tn*5398*. The CRISPR-Cas9 system developed in this study was then further exemplified via the generation of 630Δerm*Δ*pyrE*, a truncated *pyrE* deletion mutant compatible with the pre-existing allele-coupled exchange methodology. Crucially, replicates of each mutant were obtained lacking any SNPs or Indels which can arise during mutagenesis. This genome editing, performed in triplicate for each deletion target using three separate plasmids with unique crRNA retargeting sequences, was observed to be highly efficient in each instance. Observed editing efficiencies in this study were 96% for 630Δerm* and 89% for 630Δerm*Δ*pyrE*.

The efficiency of mutant generation observed here represents a higher frequency than obtained using the aTet-inducible system of McAllister and coworkers^16^ who described mutant generation in *pyrE* and *selD* at frequencies of 50 and 20%, respectively. A similar system based on a lactose inducible promoter was used to ‘knock-out’ *spo0A* (100%) and ‘knock-in’ a fluorescent *PpFbFPm* reporter gene^17^. Mutant colonies of the latter could not initially be detected and required that the primary transconjugants were subcultured overnight in liquid media and re-plated to single colonies before the presence of mutants could be demonstrated. In the experiment described, 80% were mutants. Interestingly, in both cases^16,17^ mutants were obtained in the absence of inducer indicating that neither promoter system (aTet or lactose) was sufficiently repressed under non-inducing conditions to prevent Cas9-mediated genome cleavage.

Another recent study regenerated 630Δ*erm* by deleting both *ermB* genes, as was done in this study, utilising the Cpf1 endonuclease instead of Cas9^22^. However, this CRISPR-Cpf1 mediated *ermB* deletion was performed in a lab strain of 630 and no whole genome sequencing of the subsequent mutants was reported. Hence, our 630Δerm* strain is preferable for use in future studies as we have confirmed a lack of ancillary mutations relative to the NCTC 630 reference strain.

Our developed CRISPR-Cas9 system was notably characterised by the presence in the *cas9* coding sequence of a nucleotide insertion that resulted in the premature termination of the encoded product. Despite this, our system proved highly effective, suggesting that a functional Cas9 nuclease was still being produced. The most likely explanation for this phenomenon was that re-initiation of translation occurs at a downstream, internal AUG start codon that is preceded by a sequence reminiscent of a RBS sequence. The protein (trCas9) would, therefore, be foreshortened by some 87 amino acids from the N-terminus of the native Cas9 nuclease. As the deleted region encompasses a RuvCI nucleolytic domain^23^ the variant Cas9 produced is most likely a nickase. Whilst nickase has reduced editing efficiencies compared to native Cas9, because their expression is less deleterious to the cell, larger numbers of primary transformants/transconjugant cells are obtained which, upon subsequent passages, result in mutants. As a consequence, the final total number of mutants obtained in other clostridia has been shown to be greater in comparison to the use of native Cas9^13,15^. Subsequent attempts to recreate plasmid pMTL431521 were repeated unsuccessfully, again resulting in the isolation of *cas9* variants in which various rearrangements and deletions had occurred that obviated the isolation of a fully functional *cas9* (data not shown). The most likely explanation for the repeated isolation of this variant is that the high expression of *cas9* when placed downstream of the strong P_*thl*_ promoter is detrimental to the *E*. *coli* host resulting in the selection of variants which no longer express a Cas9 nuclease with DSB activity.

The emergence of inadvertent mutations that reduce *cas9* expression underlines the importance of limiting the constitutive expression of these genes, particularly in the *E*. *coli* donor strain. Recently, a CRISPR vector for use in *C*. *sporogenes* was described^24^ in which *cas9* was placed downstream of the promoter of the *C*. *sporogenes* ferredoxin gene, P_*fdx*_. Similar to P_*thl*_, this is a strong, constitutive promoter^25^. Closer inspection of the strategy employed reveals that the 5′ end of the fragment carrying *cas9* was not cloned at an appropriate position downstream of the RBS of the P_*fdx*_ promoter of the vector used, pMTL83151. Rather the region of DNA encoding the 5′-end of *lacZα* region of pMTL83151 remained. This is predicted to result in the production of a polypeptide comprising some sixteen amino acids from LacZα (MTMITNSSSVPGDPLE) followed by a further 6 amino acids (WIRNTQ) derived from the out-of-frame fusion to the 5′-end of *cas9*, after which a TAG stop codon is encountered (Fig. S4). The *cas9* gene is not preceded by an appropriate RBS sequence. It is, therefore, highly likely that this vector exhibits Cas9 activity for the same reason as described here, through re-initiation of translation at a downstream, internal AUG start codon to produce trCas9.

*C*. *difficile* 630 is resistant to a wide range of antibiotics including Em, tetracycline and β-lactams, limiting the choice of markers which can be used in genetics studies. The previous generation of Em^S^ derivatives of 630, 630Δ*erm* and 630E, permitted the use of the *ermB* marker in ClosTron mutagenesis for the identification of successful intron insertion into the target gene. Whilst the development of CRISPR-Cas9 genome editing allows mutagenesis to be performed in wild-type strains, the availability of an Em^S^ 630 strain will still find use in studies which require multiple antibiotic resistance markers or where an *ermB* marker is to be used without risk of reversion to an Em^R^ phenotype previously observed with 630Δ*erm* at low frequencies^6^. Here we present 630Δerm*, an Em^S^ 630 strain which is sequence identical to the NCTC 13307 parental strain, except for the intended deletion within Tn*5398*.

Our initial attempts at generating a SNP-free Em^S^ strain of 630 were hampered by the presence of mutations in our laboratory stock of 630 (CRG 630) prior to the onset of this work, most notably the 21 bp deletion. Our stock of 630 was acquired from the Brendan Wren laboratory (LSHTM) in 2010, who had previously acquired it from the Mullany laboratory. A fresh stock of 630 was reacquired from LSHTM in 2018, from Lisa Dawson, and the region encompassing the deletion PCR amplified from the culture. No evidence for the presence of the deletion was evident from Sanger sequencing of the amplified DNA fragment. On receipt in 2010, the strain would have been used to prepare glycerol stocks which have been stored from that date at −80 °C until used here. It would appear that the deletion variant arose during the sub-culture undertaken to create that stock.

Since its isolation from a patient with severe pseudomembranous colitis in 1982 by Wust *et al*.^4^, *C*. *difficile* 630 was supplied to Hachler, who in turn supplied this strain to Mullany, who then supplied it to Wren, from whom we obtained our CRG 630 strain. Mullany also deposited strains 630 and the Em^S^ derivative 630Δ*erm* with the NCTC culture collection (NCTC 13307) who then supplied 630 to the DSMZ collection (DSM 27543). Given the variants accumulated in our CRG 630 strain and the differences between published 630 genomes, we reiterate our previous recommendation^11^ that bacterial strains within laboratory collections are regularly re-sequenced. Furthermore, with the ever-decreasing cost of whole genome sequencing it is essential that this is performed after the generation of mutant strains to ensure no ancillary genomic variants have been acquired along the way. A similar recommendation was made in a recent review of *C*. *difficile* 630 reference genome sequences which advocated the resequencing of all laboratory 630 strains to allow for the tracing of 630 evolution, and drew parallels between the dissemination and propagation of 630 strains in laboratories and culture collections across the world to the *E*. *coli* long-term evolution experiment^26^.

Traditional approaches to mutant generation by allelic exchange in clostridia, has been reliant on counter selection markers in a two-step selection process reliant on the initial isolation of single cross-over integrants followed by the isolation of the desired double cross-over mutants. As a consequence, repeated passages through single colony isolations are required, a process that increases the chances of isolating ancillary genomic mutations. The use of CRISPR-Cas9 allows the direct selection of clonal populations which carry the desired mutant allele, reducing the number of passages and thereby the chances of isolating inadvertent mutants. Both approaches, however, can fall foul of the need to isolate mutants that have lost the plasmid used to create them. In many instances, this can require several passages through single colony re-streaks. As demonstrated here, the more passages that are undertaken, the greater the likelihood of selecting a derivative containing SNPs or Indels. In this respect, the number of passages used to cure the CRISPR-Cas9 vector employed to create the mutant should be wherever possible minimised.

## Methods

### Bacterial strains and plasmids

All bacterial strains and plasmids utilised in this study are listed in Tables 1 and 2 respectively. *E*. *coli* was cultured in LB medium, supplemented where appropriate with chloramphenicol (25 μg.ml^−1^), at 37 °C with horizontal shaking at 200 rpm. *C*. *difficile* was cultured anaerobically at 37 °C in an anaerobic MACS1000 workstation (Don Whitely, Yorkshire, UK) in BHIS (Brain Heart Infusion supplemented with yeast extract [5 mg.ml^−1^] and L-cysteine [0.1% w/v]) medium supplemented with d-cycloserine (250 μg.ml^−1^), cefoxitin (8 μg.ml^−1^) and thiamphenicol (15 μg.ml^−1^) or Em (10 μg.ml^−1^) where appropriate. Confirmation of *pyrE* truncation was performed by plating strain onto *C*. *difficile* minimal medium described previously^27^, supplemented with uracil (20 μg.ml^−1^) and 5-flouroorotic acid (4 mg.ml^−1^) where appropriate.

**Table 1:** List of bacterial strains utilised in this study.

Bacterial Strain	Description	Source
*E*. *coli* TOP10	F-*mcrA* Δ(*mrr*-*hsd*RMS-*mcr*BC) Φ80*lac*ZΔM15 Δ*lac*X74 *recA1 araD139* Δ(*ara leu*) 7697 *galU galK rpsL* (Str^R^) *endA1 nupG*	Invitrogen
*E*. *coli* CA434	Conjugation donor	Purdy *et al*.^28^
*C*. *difficile* 630 (CRG856)	Ribotype-012 wild-type (Zurich, Switzerland)	P Mullany, UCL
*C*. *difficile* 630 (NCTC 13307)	Ribotpye-012 wild-type, reference strain (Zurich, Switzerland)	National Collection of Type Cultures (NCTC), Public Health England
*C*. *difficile* 630Δ*erm*	Em^S^ derivative of *C*. *difficile* 630 lacking *erm2*(*B*)	Hussein *et al*.^6^
*C*. *difficile* 630Δerm*	Em^S^ *C*. *difficile* 630 strain lacking *erm1*(*B*) and *erm2*(*B*)	This study
*C*. *difficile* 630Δerm*Δ*pyrE*	*C*. *difficile* 630Δerm* lacking 234 base-pairs from 3′-end of the *pyrE* gene	This study

**Table 2:** List of plasmids utilised in this study.

Plasmid	Description	Source
pMTL83151	*E*. *coli*-clostridia shuttle vector with pCB102 replicon	Heap *et al*.^19^
pMTLcas-pta	CRISPR-Cas9 genome editing vector harbouring *cas9 gene* from *Streptococcus pyogenes*	Huang *et al*.^14^
pMTL431511-CLAU-pyrE	Modular CRISPR-Cas9 vector targeting *C*. *autoethanogenum pyrE*	This study
pMTL431521-CDF-2008A	pMTL431511-CLAU-pyrE with TcdB promoter driving sgRNA expression, 2008A crRNA sequence and Tn*5398* editing template	This study
pMTL431521-CDF-2008B	pMTL431521-CDF-2008A with 2008B crRNA sequence	This study
pMTL431521-CDF-2008C	pMTL431521-CDF-2008A with 2008C crRNA sequence	This study
pMTL431521-CDF-pyrE1	pMTL431521-CDF-2008A containing *pyrE* editing template and *pyrE1* crRNA sequence	This study
pMTL431521-CDF-pyrE2	pMTL431521-CDF-pyrE1 with pyrE2 crRNA sequence	This study
pMTL431521-CDF-pyrE3	pMTL431521-CDF-pyrE1 with pyrE3 crRNA sequence	This study

### Modularisation of the CRISPR-Cas9 vector

First, *cas9* and P_*thl*_ from pMTLcas-pta were amplified using Pthl-F-XbaI and Cas9R-NotI, with the resulting PCR product digested with *Xba*I and *Not*I and cloned into a pMTL83151 backbone linearised with the same enzymes. Following Sanger sequence confirmation with CatP-R1, *pyrE* targeting sgRNA cassette was generated and inserted described below. Using pMTLcas-pta as a template, the P_*araE*_ promoter was amplified with sgRNA-F-XbaI and sgRNA-pyrE-sg3-SOE-R, with the latter primer containing the 20 nt *pyrE* targeting seed region as an overhang. Simultaneously, the sgRNA handle and its associated terminator were amplified using sgRNA-pyrE-sg3-SOE-F and sgRNA-R-MauBI-AscI primers, with the former containing the 20 nt *pyrE* targeting seed region as an overhang. Using the flanking sgRNA-F-XbaI and sgRNA-F-MauBI-AscI primers, these two PCR products were spliced via SOEing PCR using the overlapping seed region. The resulting spliced PCR product was digested with *Xba*I and *Asc*I, and cloned in the Cas9 containing pMTL83151 vector described above (also digested with *Xba*I and *Asc*I). Again, CatP-R1 was used for Sanger sequence confirmation of this intermediate vector, prior to insertion of the *pyrE* homology arm cassette, resulting in the final pMTL43151-CLAU-pyrE vector.

For homology cassette generation, primer pairs CLAU-pyrE-LHA-F-MauBI + CLAU-pyrE-LHA-R, and CLAU-pyrE-RHA-F + CLAU-pyrE-RHA-R-AscI were used to generate the *pyrE* LHA and RHA, respectively. Following this, the flanking primers CLAU-pyrE-LHA-F-MauBI and CLAU-pyrE-RHA-R-AscI were used to splice the homology arms, with the subsequent PCR product digested with *Mau*BI and *Asc*I. The digested homology arm cassette was cloned in a *Mau*BI and *Asc*I linearised backbone of the intermediate Cas9 and sgRNA containing pMTL83151 vector described above, to generate the final pMTL43151-CLAU-pyrE vector. Following the identification of issues regarding *Mau*BI digestion efficiency, the relevant primer pairs were adjusted in order to incorporate the *Asi*SI recognition site in place of the *Mau*BI site, as detailed in Supplementary Table S4. Final vector sequence confirmation was achieved using CatP-R1 and sgRNA-F-XbaI primers.

Having modularised the pMTLcas-pta into the pMTL40000 vector series as outlined in the results section, we next sought to modify this system for use in *C*. *difficile* via replacement of the P_*araE*_ promoter controlling sgRNA expression. Accordingly, the promoter region of *tcdB* was PCR amplified from *C*. *difficile* 630 genomic DNA using primer pair PtcdB_XbaI and PtcdB_SalI and cloned between *Xba*I and *Sal*I restriction sites within pMTL431511-CLAU-pyrE.

For the deletion of a 3.6 kb fragment containing the *erm1*(*B*) and *erm2*(*B*) genes from Tn*5398* an editing template for homologous recombination was generated. PCR primer pairs 630erm_asiSI_LF1 & 630erm_LR1, and 630erm_RF1 & 630erm_ascI_RR1, were used to amplify 0.977 kb and 1.025 kb homology arms, respectively, from *C*. *difficile* 630 genomic DNA. These homology arms were fused together using splicing by overlap extension PCR (SOE-PCR) with primers 630erm_asiSI_LF1 and 630erm_ascI_RR1 and the resulting editing template cloned into pMTL431511-CLAU-pyrE between *Asi*SI and *Asc*I restriction sites. To retarget the sgRNA submodule of the resulting vector, we identified 20-nucleotide guide retargeting sequences upstream of 5′-NGG-3 PAMs within the 3.6 kb chromosomal deletion fragment using the CRISPR guide design tool on Benchling (www.benchling.com)^20^. Three guide sequences were selected with the highest on-target and off-target scores, across both DNA strands and incorporated into oligonucleotides with flanking 25-nucleotide regions of overlapping homology to the 3′-end of P_*tcdB*_ and 5′-end of the sgRNA handle. These oligonucleotides were separately incorporated into *Sal*I-linearized vector using HiFi DNA assembly (NEB), the assembly reaction products were transformed into *E*. *coli* DH5α and the Sanger sequencing confirmed vectors named pMTL431521-CDF-2008A, pMTL431521-CDF-2008B and pMTL431521-CDF-2008C.

Similarly, for generating truncated *pyrE* mutants in *C*. *difficile* 630 lacking 234 bp from the 3′ end of the gene, 1.338 and 1.026 kb homology arms were PCR amplified using primer pairs 630pyrE_asiSI_LF1 & 630pyrE_LR1, and 630pyrE_RF1 & 630pyrE_ascI_RR1, respectively, and fused together using SOE-PCR with primers 630pyrE_asiSI_LF1 and 630pyrE_ascI_RR1. The resulting editing template was cloned between *Asi*SI and *Asc*I sites of pMTL431521-CDF-2008A. Next, this vector was linearized with *Sal*I as previously to allow replacement of guide targeting sequences using HiFi assembly with oligonucleotides pyrE1_HiFi, pyrE2_HiFi and pyrE3_HiFi generating the vectors pMTL431521-CDF-pyrE1, pMTL431521-CDF-pyrE2 and pMTL431521-CDF-pyrE3.

### CRISPR-Cas9 mutagenesis

CRISPR-Cas9 vectors were transferred to *C*. *difficile* 630 strains via conjugation from *E*. *coli* CA434 donors as described previously^28^. Thiamphenicol resistant transconjugant colonies appeared within 72 hours and were streaked to purity. Resulting single colonies were used as the template in colony PCR screens for the desired deletions using primer pairs annealing to chromosomal regions flanking the editing template sequences, Tn5398_sF1 & Tn5398_sR1 for 630Δerm* and CD630_pyrD_sF1 & CD630_0189_sR3 for 630Δerm*Δ*pyrE*. Colony PCR products were separated using agarose gel electrophoresis.

### Illumina Sequencing and bioinformatics

Genomic DNA was isolated from *C*. *difficile* overnight cultures using phenol-chloroform extraction and stored in Tris-HCl buffer (10 mM, pH 7.8). *C*. *difficile* genomic DNA was sequenced using the Illumina MiSeq platform (DeepSeq, Nottingham, UK) using 500 bp V2 SBS chemistry. Analysis of the generated reads and identification of single nucleotide polymorphisms, insertions and deletions was performed using the Basic Variant Detection tool within CLC Genomics Workbench (version 11.0.1) by mapping the trimmed Illumina paired-end reads to the reference sequences AM180355.1^3^, CP010905.2^21^, LN614756^29^ or CP016318^30^. Variants were detected with minimum coverage, count and frequency of 10, 2 and 70%, respectively, with base quality filter settings of neighbourhood radius = 5, minimum central quality = 20 and minimum neighbourhood quality = 15, applied.

### Development of heat-resistant CFU over 5 days

Sporulation assays observing the development of heat resistant CFU over 5 days were performed as described previously^31,32^. Briefly, sporulation cultures, including a 630Δ*erm*Δ*spo0A* sporulation negative control^12^, were grown in triplicate in BHIS broth for 5 days, with two 500 µl samples taken after 0, 24, 48, 72, 96 and 120 hours. From each time-point, one sample was heat-treated (65 °C, 30 minutes) whilst the other was incubated on the bench. Following incubations, samples were serially diluted from 10^0^ to 10^−7^ in PBS and three 20 µl aliquots of each dilution were spotted onto BHIS agar supplemented with 0.1% taurocholic acid. After 24 hours, colonies were counted and CFU.ml^−1^ values determined.

### MIC determinations

Determinations of the minimum inhibitory concentration of *C*. *difficile* strains to Em was performed using the broth microdilution method described previously^33^. Overnight *C*. *difficile* cultures were sub-cultured into pre-reduced BHIS broth supplemented with Em concentrations ranging from 0 to 64 ug.ml^−1^. After 24 hours incubation, OD_600_ measurements were performed using a GloMax-Multi Microplate Multimode Reader (Promega, USA) and the MICs determined.

## Supplementary information

Supplementary Information
