# Supplementary material for: Generation of a fully erythromycin-sensitive strain of Clostridioides difficile using a novel CRISPR-Cas9 genome editing system
Source: Sci Rep. 2019 May 31;9:8123. doi: 10.1038/s41598-019-44458-y (PMC6544763; doi:10.1038/s41598-019-44458-y)
Supplement: Supplementary file 1 — Supplementary Information [file 41598_2019_44458_MOESM1_ESM.pdf]

Supplementary Material

Generation of a fully erythromycin-sensitive strain of *Clostridioides difficile* using a CRISPR-Cas9 genome editing system.

Patrick Ingle[1], Daphne Groothuis[1], Peter Rowe[1], He Huang[3], Alan Cockayne[1], Sarah A Kuehne[2], Weihong Jiang[3], Yang Gu[3], Christopher M Humphreys[1], Nigel P Minton[1]

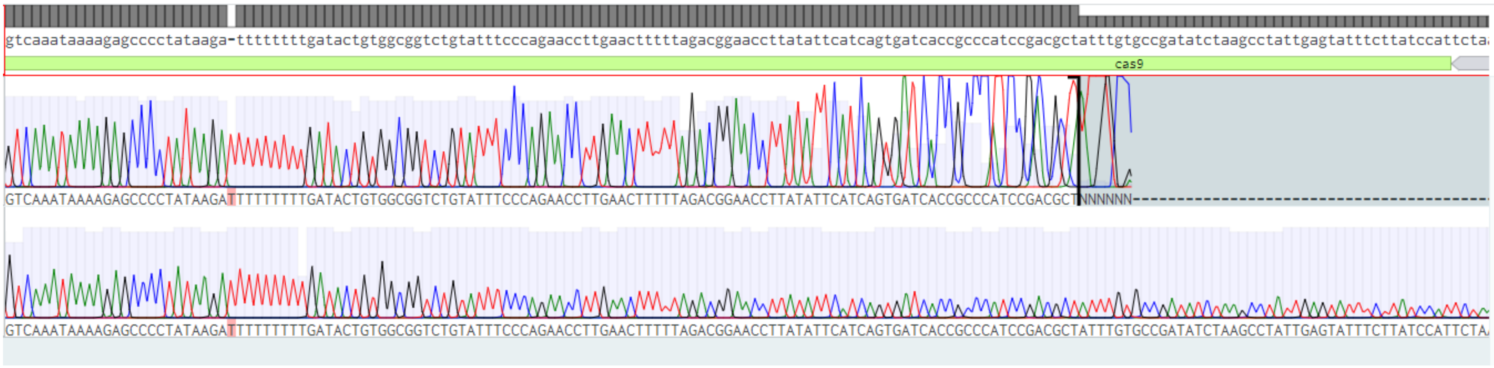

Figure S1. Chromatograph of the frameshift mutation in the *cas9* gene of plasmid pMTLcas-pta. At the time of its construction at the Shanghai Institute of Biological Sciences (SIBS), no mutations were noted in the *cas9* gene of the plasmid. The plasmid DNA used for this Sanger sequencing was sent to Nottingham, where it was used to transform *E. coli* and a random, single colony selected for all subsequent work. Sanger sequencing of the plasmid carried by this transformant subsequently revealed the presence of the frame-shift mutation, explaining its presence in all derivative plasmids made in the current study. The likelihood is that a proportion of the original SIBS clone is composed of a sub-population carrying the pMTLcas-pta plasmid variant containing this mutation, and that this plasmid was selected during the transformation experiment at Nottingham. The factors that influenced this occurrence, other than chance, are unknown. Nonetheless, the emergence of this variant emphasises the need to avoid placing *cas9* downstream of strong constitutive promoters in the *E.coli* shuttle host.

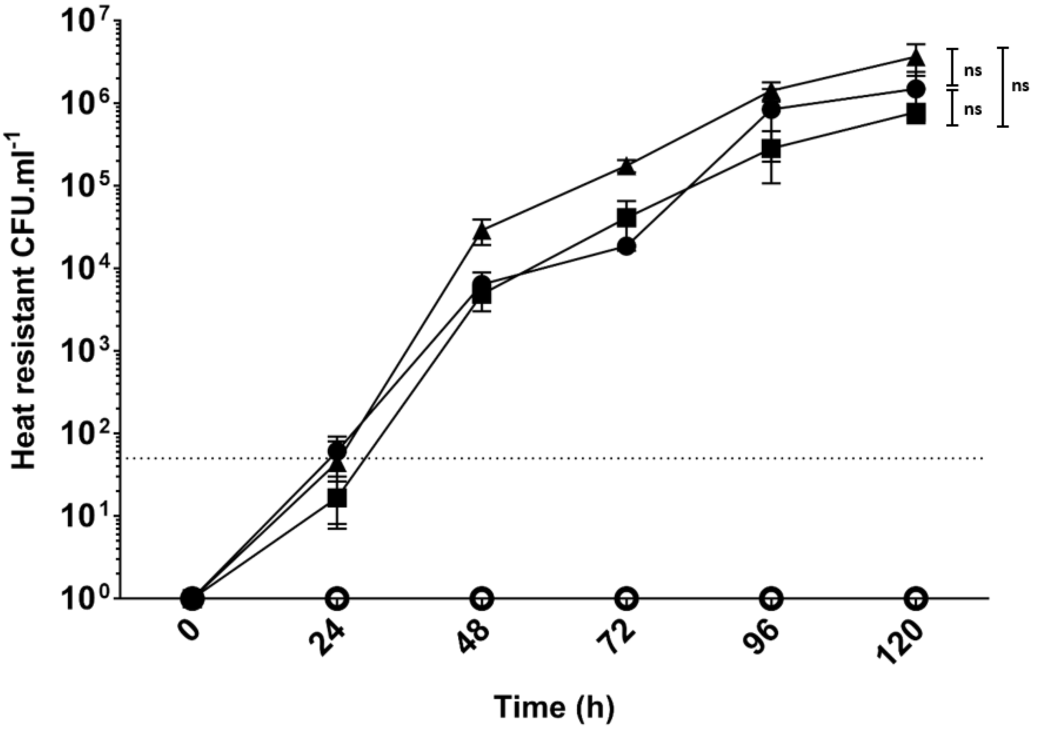

Figure S2. Development of heat resistant CFU of *C. difficile* 630 strains over five days. ●, *C. difficile* 630; ▲, *C. difficile* 630Δ*erm*; ■, *C. difficile* 630Δ*erm**; **O**, *C. difficile* 630Δ*erm*Δ*spo0A*. Symbols represent the mean values from three independent experiments. Error bars indicate standard errors of the means. The dotted line at 50 CFU.ml^{-1} denotes the detection limit for the assay. Statistical significance determined using one-way ANOVA with Dunnett's multiple comparisons test. Not significant (ns) denotes a *P*-value >0.05.

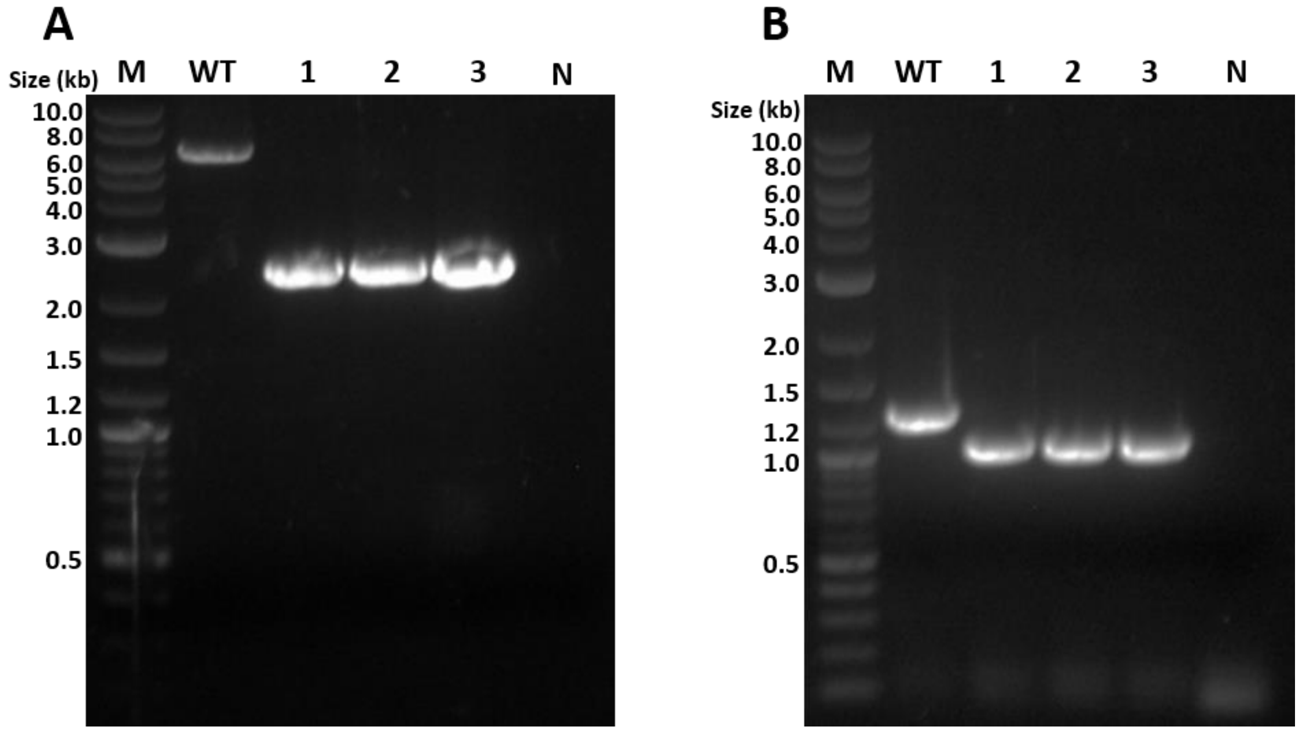

Figure S3. Regeneration of 630Δ*erm** and 630Δ*erm**Δ*pyrE* in the newly acquired 630 (NCTC 13307) reference strain. Agarose gel electrophoresis following colony PCR screens of three independently generated NCTC 630Δ*erm**Δ*pyrE* CRISPR-Cas9 mutants (lanes 1-3). A) Confirmation of the deletion of a 3.6 kb fragment containing both *erm(B)* genes using screening primers Tn5398_sF1 and Tn5398_sR1. B) Confirmation of a 234 bp deletion in *pyrE* using screening primers CD630_pyrD_sF1 & CD630_0188_sR1. 'M' denotes DNA marker, 'WT' denotes wild-type NCTC 13307 genomic DNA and 'N' denotes the negative control using dH_2O as template.

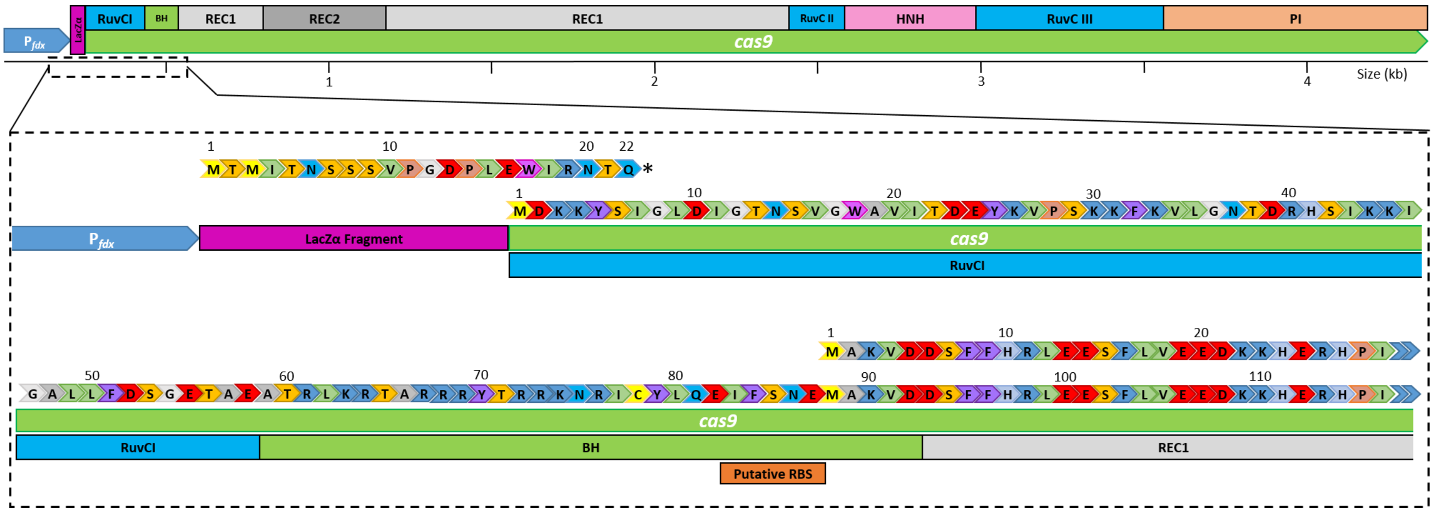

Figure S4. Diagrammatic illustration of the sequence and annotated features of pMTL83153-cas9 from Guo *et al.* [24] used to genome edit *Clostridium sporogenes*. Top: Domain organisation within wild-type *S. pyogenes cas9*. Bottom: Domain organisation and amino acid sequence of a trCas9 resulting from the cloning of *S. pyogenes cas9* into pMTL83153 at *Xba*I and *Xho*I sites downstream of the ferrodoxin promoter from *C. sporogenes* (P_{fdx}). This leaves a 47 bp lacZα fragment between P_{fdx} and *cas9* resulting in a transcript of 22-amino acids being produced, with a premature stop codon (*) present early within the RuvCI domain of *cas9*. A putative ribosome binding site and downstream trCas9 ORF lacking 87 amino acids from the N-terminal end of Cas9 proposed in our study (Fig. 4) are also indicated.

			CRG 630			CRG 630Δ*erm**rep 1			CRG 630Δ*erm** rep 2			CRG 630Δ*erm** rep 3			
Position	Type	Ref.	Allele	Cov	Freq	Allele	Cov	Freq	Allele	Cov	Freq	Allele	Cov	Freq	Gene (AA change)
103225	SNV	G	T	40	100	T	85	100	T	72	100	T	60	100	*rplC*
268934	SNV	G	T			T	64	98	T	62	98	T	51	98	CD630_02050 (G165C)
690658	SNV	A	T	38	100	T	59	100	T	45	100	T	52	100	IG
1391850	SNV	T	C	32	100	C	60	100	C	42	100	C	44	100	CD630_11900 (F133L)
1607453	INS	-	T	36	91.7	T	56	91	T	39	97	T	46	85	CD630_13880
2044514	SNV	C	G	32	100	G	56	100	G	38	100	G	30	100	*gapB* (P33A)
2203033	SNV	A	T	36	97.2	T	48	100	T	35	100	T	30	100	IG
2832892	SNV	G	T	21	100	T	50	100	T	57	100	T	22	100	IG
2924655	SNV	C	T	23	100	T	70	100	T	60	100	T	30	100	CD630_25320
3034953	SNV	C	A	34	100	A	65	100	A	61	100	A	51	100	CD630_26270 (G68C)
3080703	SNV	C	T	28	100	T	65	100	T	60	100	T	53	100	*ptsG-BC* (V228I)
3105406	DEL	21 nt	-	13	100	-	29	100	-	30	100	-	18	100	CD630_26850 (ΔE115-A121)
3590230	SNV	T	G	38	100	G	90	100	G	19	100	G	19	100	CD630_30890 (E258D)
3686535	INS	-	A	52	100	A	49	98	A	22	100	A	20	100	CD630_31561 (L104fs)
3797112	SNV	C	T	63	98.4	T	82	100	T	57	98	T	46	100	*prdR* (E261K)
4007463	INS	-	C	26	100	C	12	100	C	61	100	C	45	100	IG
4007603	SNV	A	G	27	100	G	15	100	G	70	97	G	63	100	IG

Table S1. Identification of genomic variants in CRG 630 and CRG 630Δ*erm** strains following Illumina whole genome sequencing. Single nucleotide variants (SNVs), insertions (Ins) and deletions (del) detected in the CRG 630 parental strain and three independent 630Δ*erm** mutant strains generated via CRISPR-Cas9 mutagenesis, compared with the CP010905.2 630 reference sequence [21]. Variant alleles, their coverage (Cov) and frequency (Freq), are shown at the stated position in the reference sequence. Annotations at each variant locus are listed in the 'Gene' column, with any non-synonymous mutations indicated in parentheses.

			NCTC 13307			630Δ*erm** rep 1			630Δ*erm** rep 2			630Δ*erm** rep 3			
Position	Type	Ref	Allele	Cov	Freq	Allele	Cov	Freq	Allele	Cov	Freq	Allele	Cov	Freq	Gene (AA change)
97003	SNV	A	G	112	100	G	152	100	G	58	100	G	113	100	*rpoB* (K517E)
854723	Ins	-	A	106	94.34	A	149	88.59	A	75	87.21	A	126	93.65	Non-coding region
895022	SNV	C							A	70	100				CDIF630_00864 (P37T)
985682	SNV	T	G	106	100	G	138	100	G	56	100	G	121	100	*perR* (V62G)
985742	SNV	G				T	125	100							*perR* (C82F)
1234305	SNV	C							T	47	100				*eftA3* (Q256STOP)

			NCTC 13307			630Δ*erm**Δ*pyrE* #1			630Δ*erm**Δ*pyrE* #2			630Δ*erm**Δ*pyrE* #3			
Position	Type	Ref	Allele	Cov	Freq	Allele	Cov	Freq	Allele	Cov	Freq	Allele	Cov	Freq	Gene (AA change)
97003	SNV	A	G	112	100	G	111	100	G	139	100	G	189	100	*rpoB* (K517E)
854723	Ins	-	A	106	94.34	A	146	93.15	A	121	95.04	A	173	93.06	Non-coding region
895022	SNV	C							A	136	99.26				CDIF630_00864 (P37T)
985682	SNV	T	G	106	100	G	138	100	G	106	99.06	G	190	100	*perR* (V62G)
985742	SNV	G				T	125	100							*perR* (C82F)
1234305	SNV	C							T	89	100				*eftA3* (Q256STOP)

Table S2. Variant detection following whole genome sequencing of three independently generated 630Δ*erm** and 630Δ*erm**Δ*pyrE* strains. Single nucleotide variants (SNVs) and insertion (Ins) detected in the newly acquired 630 reference strain NCTC 13307, three 630Δ*erm** and three 630Δ*erm**Δ*pyrE* strains, independently generated via CRISPR-Cas9 mutagenesis, when compared with the CP010905.2 630 reference sequence [21]. Variant alleles, their coverage (Cov) and frequency (Freq), are shown at the stated position in the reference sequence. Annotations at each variant locus are listed in the 'Gene' column, with any non-synonymous mutations indicated in parentheses. Additional variants to those found in the parental 630 NCTC 13307 strain are highlighted in red.

			AM180355.1			CP010905.2			LN614756.1			CP016318.1			
Position	Type	Ref	Allele	Cov	Freq	Allele	Cov	Freq	Allele	Cov	Freq	Allele	Cov	Freq	Gene
84143	SNV	C	T	20	100										*tuf1*
97003	SNV	A				G	112	100							*rpoB*
103225	SNV	G	T	113	100										*rplC*
612577	SNV	A										C	62	83.9	Non-coding
612783	SNV	G										T	65	84.6	Non-coding
682619	SNV	C	T	52	73.1										Non-coding
690658	SNV	A	T	109	100										Non-coding
854723	INS	-				A	106	94.3							Non-coding
985682	SNV	T				G	106	100							*perR*
1006274	SNV	G							A	96	100	A	96	100	*perR*
1141519/ 1146798	SNV	A	G	75	100				G	75	100	G	75	100	*ubiA*/CD630_09670
1141554/ 1146833	SNV	G	T	87	100				T	87	100	T	87	100	*ubiA*
1141559/ 1146838	SNV	T	C	85	100				C	85	100	C	85	100	*ubiA*
1391850	SNV	C							T	99	100	T	99	100	CDIF630erm_01339
1607453	Ins	-	T	91	94.5										CD630_13880
1876735- 1876933	32 VARS								32*						
2044514	SNV	C	G	77	98.7										*gapB*
2162757	SNV	T							A	96	97.9	A	96	97.9	Non-coding
2168960	SNV	A							G	81	100	G	81	100	*eutG*
2634897/ 2592214	SNV	C	T	77	98.7				T	77	98.7	T	77	98.7	CD630_22730
2832892	SNV	G	T	87	100										Non-coding
2894493	SNV	A							C	94	100	C	94	100	Non-coding
2924655	SNV	C	T	77	98.7										CD630_25320
2963183	SNV	G							T	95	100	T	95	100	Non-coding
2992270	SNV	A							C	94	100	C	94	100	CDIF630erm_02881
3038020	SNV	T							C	109	100	C	109	100	*ptsG-BC*
3528665/ 3485885	SNV	G	T	104	100				T	104	100	T	104	100	Non-coding
3548101	SNV	T										A	93	98.9	CDIF630erm_03372
3548323	SNV	A							G	125	100	G	125	100	Non-coding
3686535	INS	-	A	124	98.4										CD630_31561
3905078	SNV	A										C	116	100	*mgtA2*
4007463	INS	-	C	18	100										Non-coding
4007603	SNV	A	G	27	70.4										Non-coding
4169291	SNV	A							G	133	100	G	133	100	CDIF630erm_03885

Table S3; Comparison of *C. difficile* 630 reference sequences. Genomic variant detection was performed following alignment of NCTC 630 Illumina sequencing reads against various *C. difficile* 630 (AM180355.1 [3], CP010905.2 [21]) or *C. difficile* 630Δ*erm* (LN614756.1 [29], CP016318.1 [30]) reference sequences. Variant alleles, their coverage (Cov) and frequency (Freq), are shown at the stated position in the reference sequence.

Oligonucleotide	Sequence (5'-3')	Description
Pthl-F-XbaI	ATATATTCTAGATTTTAACAAAATATATTGATAAAAATAATAATAGTG	Amplification of *cas9* gene and thiolase promoter from pMTLcas-pta
Cas9R-NotI	ATATATATGCGGCCGCTCAGTCACCTCCTAGCTGACTC	
CatP-R1	CAAGTTTATCGCTCTAATGAAC	Sequencing primer
sgRNA-F-XbaI	ATATATTCTAGATTTATATTTAGTCCCTTGCC	Amplification of the P_{araE} promoter from pMTL-cas-pta
sgRNA-pyrE-sg3-SOE-R	TTTGCCTTGCAAGTTCATAGGTCGACTCTAGAGGATCCC	
sgRNA-pyrE-sg3-SOE-F	CTATGAACTTGCAAGGCAAAGTTTTAGAGCTAGAAATAGCAAG	Amplification of sgRNA handle and terminator region from pMTL-cas-pta
sgRNA-R-MauBI-AscI	ATATATCTCGAGATATATCGCGCGCGATAAAAATAAGAAGCCTGCAAATG	
CLAU-pyrE-LHA-F-MauBI	ATATATATCGCGCGCGTAAATTTAGGTGAAGAACACCATACATATCC	Amplification of left and right homology arms and SOEing PCR to generate the editing template targeting the *C. autoethanogenum pyrE* gene
CLAU-pyrE-LHA-R	ATAATTTAGGAGGACAGTTATGGATAAATAAGTCGAAAAAATCAATGCACGATGCAGAATTGAC	
CLAU-pyrE-RHA-F	GTGCATTGATTTTTTCGACTTATTTATCCATAACTGTCCTCCTAAATTATTCCTCTTATTTCAC	
CLAU-pyrE-RHA-R-AscI	ATATATATGGCGCGCCATATAGTACTGTAATTTTTAAAAAGTTTGAAATAACTAGTTAG	
PtcdB_*Xba*I	ATATATTCTAGATTAATGAATTTAAAGAAATATTTACAATAG	Amplification of *C. difficile tcdB* promoter for replacement of P_{araE} in pMTL431521 vectors
PtcdB_*Sal*I	ATATATCAGCTGATTTTCTCCTTTACTATAATATTTTTATTG	
630erm_asiSI_LF1	ATATATGCGATCGCAACCATTCTTACCGCATTGC	Amplification of left and right homology arms and SOEing PCR to generate the editing template to delete 3.6 kb from within *C. difficile* 630 Tn*5398*.
630erm_LR1	TATCATCAACAATCACAGTAATCACTCCTGAAGTGATTACATC	
630erm_RF1	TTACTGTGATTGTTGATGATAAAATAAGAATAAGAAG	
630erm_ascI_RR1	ATATATGGCGCGCCATCGTCCTTGTGAAGCACAAGC	
CD2008A_HiFi	TTATAGTAAAGGAGAAAATGTCGACAACATCAAATCAAACGTGCCGTTTTAGAGCTAGAAATAGCAAGTT	Insertion of crRNAs targeting CD630_2008 into pMTL431521-CDF-2008A via HiFi assembly
CD2008B_HiFi	TTATAGTAAAGGAGAAAATGTCGACTTGCAAGAACAGTTTAACCCGTTTTAGAGCTAGAAATAGCAAGTT	
CD2008C_HiFi	TTATAGTAAAGGAGAAAATGTCGACAATGGCATTACAGAACACAAGTTTTAGAGCTAGAAATAGCAAGTT	
Tn5398_sF1	ATACGTCTTATTTCCCAGATATGC	Colony PCR screening of putative 630Δ*erm** mutants
Tn5398_sR1	TCTGATGGCGTTTCATAAGC	
630pyrE_*asi*SI_LF1	ATATATGCGATCGCTTAAGTGTGAAATTTGGAAGTGTAG	Amplification of left and right homology arms and SOEing PCR to generate the editing template to remove 234 bp from the 3'-end of *C. difficile* 630 *pyrE*.
630pyrE_LR1	AAGTTTTTATTAAACGTCTTCAGCAATTATTATCTTTGC	
630pyrE_RF1	TAATTGCTGAAGACGTTTAATAAAAACTTAATTATTTATAGTGTTACTTAAAAAATG	
630pyrE_*asc*I_RR1	ATATATGGCGCGCCATCTTGAAGCATTGATGTTCTTCC	
pyrE1_HiFi	TTATAGTAAAGGAGAAAATGTCGACAGAGTATTAGAAGCCTTAGGGTTTTAGAGCTAGAAATAGCAAGTT	Insertion of crRNAs targeting CD630_0187 into pMTL431521-CDF-pyrE1 via HiFi assembly
pyrE2_HiFi	TTATAGTAAAGGAGAAAATGTCGACGAGTGTCCTTTATGTAAGGAGTTTTAGAGCTAGAAATAGCAAGTT	
pyrE3_HiFi	TTATAGTAAAGGAGAAAATGTCGACACCTACAACTTCTCCACCTAGTTTTAGAGCTAGAAATAGCAAGTT	
CD630_pyrD_sF1	AGAGAAGGAATAAAAAGTTTAGACGAAATAAGAGG	Colony PCR screening of putative 630Δ*erm**Δ*pyrE* mutants
CD630_0189_sR3	CCAAGCTCTATGACAGACAGCTCATTGTTTAGAAC	
CD630_0188_sR1	AATCTTTCTCCTATTATCTCTTCG	

Table S4; List of oligonucleotide primers utilised in this study.
